# Zebrafish *cdh23* Affects Rod Cell Phototransduction Through Regulating Ca^2+^ Transport and MAPK Signaling Pathway

**DOI:** 10.3390/ijms26104604

**Published:** 2025-05-11

**Authors:** Xiaoying Zheng, Binling Xie, Dingrui Chen, Jifan Jiang, Ting Zeng, Lei Xiong, Qingying Shi, Hao Xie, Yisheng Cai, Jiaxin Liang, Song Chen, Xiaochao Qu, Huaping Xie

**Affiliations:** 1 Laboratory of Animal Nutrition and Human Health, Hunan International Joint Laboratory of Animal Intestinal Ecology and Health, College of Life Science, Hunan Normal University, Changsha 410081, China; 202370142927@hunnu.edu.cn (X.Z.); icxbl1@hunnu.edu.cn (B.X.); 202230195064@hunnu.edu.cn (D.C.); casjjf@163.com (J.J.); tingz@hunnu.edu.cn (T.Z.); xl@hunnu.edu.cn (L.X.); 19118764408@163.com (Q.S.); haox@hunnu.edu.cn (H.X.); 17680154624@163.com (J.L.); 2 Laboratory of Molecular and Statistical Genetics, College of Life Sciences, Hunan Normal University, Changsha 410081, China; cystao@csu.edu.cn (Y.C.); quxc@hunnu.edu.cn (X.Q.); 3 School of Medicine, Hunan Normal University, Changsha 410081, China; chensong@hnsfdxxyzh.wecom.work

**Keywords:** photoreceptor degeneration, apoptosis, *CDH23*, gene knockout, Ca^2+^, MAPK

## Abstract

Mutations in the pathogenic gene *CDH23* are known to cause Usher syndrome, affecting both auditory and visual functions. Our previous results provided valuable insights into the mechanisms underlying congenital hearing loss associated with *CDH23* mutations. However, the molecular mechanisms and signaling pathways that influence vision remain largely unknown. In this study, transcriptional sequencing and bioinformatics analysis were conducted to compare gene expression between the control and *cdh23^−/^*^−^. Additionally, RT-qPCR experiments were performed to further validate the bioinformatics analysis results. The comparative transcriptomic analysis identified differentially expressed genes associated with photoreceptor degeneration and the mitogen-activated protein kinase (MAPK) signaling pathway. Embryos were subjected to hematoxylin and eosin (H&E) staining to assess their histological changes. The results showed that the *cdh23^−/−^* retina was morphologically indistinguishable from the control. Apoptosis was assessed using TUNEL staining, which revealed an increase in total cell death in the *cdh23^−/^*^−^ retina. Our results revealed that the cell death was induced by Ca^2+^ and MAPK signaling interactions following photoreceptor degeneration. This study provides insights into the mechanisms underlying the role of *cdh23* in vision.

## 1. Introduction

Cadherins are a superfamily of calcium-dependent cell adhesion molecules, which are widely expressed in living tissues. In the retina and retinal pigment epithelium (RPE), cadherins play crucial roles in tissue morphogenesis, the formation of neural circuits, the establishment of adhesion junctions in the outer blood–retina barrier, photoreceptor disk morphogenesis, maintenance, and survival. Cadherin-23 (CDH23) is a non-classical cadherin protein that belongs to the cadherin superfamily [1], which has been identified as a genetic cause of hereditary retinal degeneration, i.e., the retinal cadherinopathies [2]. Human CDH23 is located on chromosome 10 and encodes a protein comprising 3354 amino acids, also known as Otocadherin [3]. It is characterized by 27 extracellular cadherin domains, a single transmembrane domain, and a cytoplasmic domain [3,4]. Extracellular domains include cadherin-specific domains such as LRDE, DXD, and DXNDN [5].

To date, three different isoforms of CDH23, A, B, and C, have been described [4,6,7,8]. CDH23A contains 3354 amino acids and is the longest, with a total of 27 extracellular Ca^2+^-binding domains [9]. The splice variants of the CDH23A and B subtypes differ in the presence (A, CDH23 (+68)) or absence (B, CDH23 (−68)) of exon 68, which encodes an insertion in the cytoplasmic domain [4,6,8]. The CDH23 (+68) A is primarily expressed in the inner ear sensory epithelium, while the CDH23 (−68) B is widely expressed in the heart, kidney, spleen, and neural retina [3,8]. Both CDH23 (+68) A and CDH23 (+68) B ensure the binding of stereocilia through a tip link in unison with protocadherin 15 in the inner ear. They also contribute to the shape of the outer segment through their expression in the inner and outer segments, as well as in the calyceal processes [10,11,12]. The later-identified short CDH23 subtype C consists only of a cytoplasmic domain [13].

In the inner ear, cadherin-23 is a key component of the tip link in stereocilia bundles [14], playing a crucial role in hair cell mechanotransduction [15]. In the retina, CDH23 is located in the inner segment, connecting cilia, and basal body complex, as well as the ribbon synapses of rod and cone photoreceptor cells. The EC domains in the transmembrane form of CDH23 (+68) are thought to be involved in mediating intermembrane adhesions between the inner segment membranes of adjacent photoreceptor cells, as well as between the presynaptic and postsynaptic membranes of photoreceptor cells and second-order retinal neurons. In synapses, CDH23 (+68) is believed to keep the synaptic cleft at a close distance, which is conducive to the organization of presynaptic and postsynaptic cytomatrices of a synaptic junction, and plays an important role in synaptogenesis [9,16,17,18]. Mutations in this calcium-binding protein are associated with Usher syndrome [19,20] and retinitis pigmentosa (RP) [21,22].

Usher syndrome is a common autosomal recessive disorder, which is classified into three subtypes according to the severity of hearing loss and photoreceptor degeneration [23]. Furthermore, UShel type I (USH1) is the most severe form, accounting for approximately 25–44% of all Usher syndrome cases [9,20,22,24,25]. Each subtype of Usher syndrome is genetically heterogeneous, and many associated genes and loci have been identified or localized, such as myosinVIIa, cadherin-23, protocadherin-15, harmonin, and SANS [26]. Nonsense mutations, frameshift mutations, splice site mutations, and some missense mutations in CDH23, all of which may result in loss-of-function alleles, lead to USH1D [23]. In 2001, three independent and nearly simultaneous reports identified CDH23 as the causative gene for USH1D [3,4,27]. USH1D is a genetic disorder characterized by congenital sensorineural hearing loss and progressive retinitis pigmentosa (RP). RP is an inherited retinal neurodegenerative disease that causes rod cell degeneration, progressive rod photoreceptor cell death, and retinal pigment epithelial cell (RPE) atrophy [28,29,30,31]. The clinical manifestations of RP in USH1D include night blindness, followed by photophobia, the gradual constriction of the visual field, and eventually total blindness [32,33].

Mutations of *MYO7A* are the most common cause of USH1 (USH1B), while *CDH23* mutations are the second most common cause of USH1 (USH1D) [29]. Evidence suggests that the mouse *myo7aa^−/−^* shaker-1 mutants exhibit mislocalized light-inducible proteins at the photoreceptor junction of the cilia [34]. The subsequent findings of a study on zebrafish *myo7aa^−/−^* mutants revealed rod cell degeneration, and consistent with the shaker-1 phenotype, an accumulation of rhodopsin was observed near the connecting cilia [35]. These results suggest that the USH1 protein may play a role in regulating protein transport within and between photoreceptors.

Several studies have been conducted on *cdh23*-related deafness. Our previous study showed that the loss of *cdh23* leads to defects in purine metabolism, resulting in ATP deficiency. This defect impairs the normal function of inner ear hair cells and causes auditory dysfunction in zebrafish [36]. However, the molecular function of *cdh23* in primate photoreceptors remains unknown. Recent evidence increasingly supports the notion that five USH1 proteins are involved in the overall molecular interaction mechanism of rod cells [11]. Moreover, the co-localization of SANS, MYO7A, and CDH23 within the connecting cilia suggests that these proteins play specific roles in the ciliary function of photoreceptors [37]. Based on these findings, we propose that CDH23 and MYO7A may exhibit similar functional characteristics.

Vision is considered one of the most important forms of human perception. In the eye, light must pass through the cornea, lens, and vitreous and retinal cell layers to reach the photoreceptor cells (rods and cones) [38,39]. These cells are responsible for receiving light stimuli and converting them into neural electrical signals, a process dependent on fluctuations in intracellular and extracellular Ca^2+^ concentrations [40]. Despite their morphological differences, the rod and cone photoreceptor cells have similar apical structures: the light-sensitive outer segment, which consists of tightly packed membranous disks, and the inner segment, which contains all the organelles required for energy production and protein synthesis. These two segments are connected by a connecting cilium [22,38,39]. A key feature of photoreceptor cells is the daily renewal of the membrane disk in the outer segment [41]. Specifically, the photoconductive proteins are continuously synthesized in the inner segment and transported to the outer segment via the connecting cilium. Degradation of the photoreceptors can lead to permanent vision loss [41,42].

Zebrafish, the widely used model organisms, have become a valuable tool in the study of both central nervous system and eye diseases [43]. Their genetic similarity to humans and their ability to regenerate damaged tissues make them ideal model organisms for genetic research. With the advancements in gene editing technology, zebrafish have increasingly been used to study a variety of eye diseases [44,45], including congenital defects, glaucoma, cone–rod dystrophy, and diabetic retinopathy [46,47]. Thus, the zebrafish *cdh23^−/−^* mutant is a useful animal model for studying RP in humans with USH1D.

This study investigated the effect of *cdh23* (F0–1 offspring have a 34 bp deletion) on the early development and differentiation of vestibular hair cells using RNA-seq technology [36]. Additionally, a systematic bioinformatics analysis was conducted to identify key gene pathways. We further checked the morphology of the retinal tissue using hematoxylin and eosin (H&E) staining, performed the TUNEL assay to detect apoptosis, and validated the expression levels of the identified genes using quantitative PCR. This study sheds light on the mechanisms by which mutations in *cdh23* contribute to sensory neural damage induced by light stimulation. Furthermore, it reveals the mechanism by which *cdh23* gene mutation contributes to the degeneration of rod photoreceptors. The study findings are significant for future research on gene diagnosis and gene therapy in the context of inherited sensory disorders.

## 2. Results

### 2.1. Histological Analysis of Retina in cdh23^−/−^ Zebrafish Mutants

**Figure 1:**
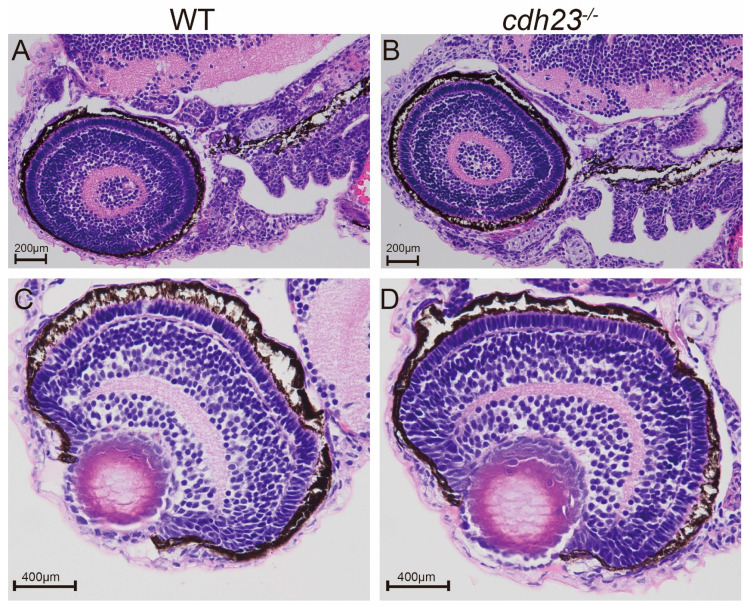
(**A**–**D**) Hematoxylin and eosin (H&E) staining. (**A**,**B**) Histological longitudinal sections of retinas from 3 dpf wild-type and *cdh23^−/−^* embryos showed no significant changes in overall retinal morphology. (**C**,**D**) Histological transverse sections of retinas from 3 dpf wild-type and *cdh23^−/−^* embryos showed no significant changes in overall retinal morphology. (**A**,**B**) Scale bar = 200 μm; (**C**,**D**) scale bar = 400 μm.

The results of startle response experiments, transcriptome data analysis, and ATP supplementation experiments that partially restored the *cdh23* defect showed that the loss of *cdh23* led to impaired metabolism and ATP deficiency, ultimately affecting the normal auditory function of zebrafish [36]. Given that *cdh23* loss causes Usher syndrome, an autosomal recessive genetic disorder characterized by both hearing loss and retinal pigment degeneration, we wondered whether the downstream effects following from the loss of *cdh23* led to visual impairment. The histology of *cdh23^−/−^* mutants at 3 dpf (Figure 1B,D) showed normal cellular organization when observed using a light microscope. At 3 dpf, the *cdh23^−/−^* retinas were morphologically indistinguishable from the control (Figure 1A,C). This finding is consistent with the previous results for *myo7aa*^−/−^ zebrafish mutants. Specifically, the histological analysis of the retina in *myo7aa*^−/−^ zebrafish at both 5 and 10 days revealed a normal retinal architecture when it was examined under the light field [35].

### 2.2. Analysis of Differential Gene Expression Between Control and cdh23^−/−^

**Figure 2:**
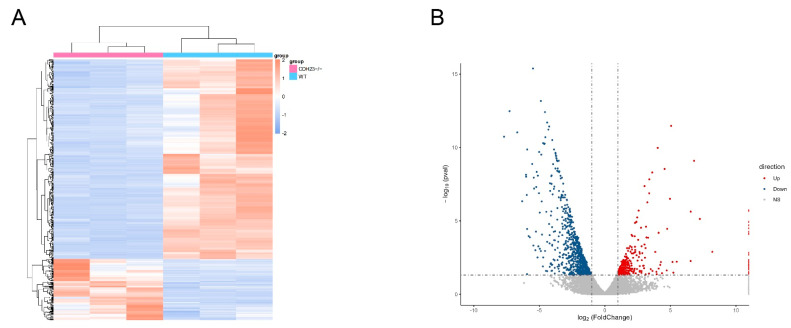
Transcriptome analyses of *cdh23^−/−^*. (**A**) Differential gene clustering heatmap: the *x*-axis represents the sample names and hierarchical clustering results, while the *y*-axis represents the hierarchical clustering results of differential genes. The red color represents up-regulated expression genes, and the blue color represents down-regulated expression genes. (**B**) Volcano plot of differentially expressed genes: the *x*-axis represents |log_2_ (foldchange)|, and the *y*-axis represents −log_10_ (*p* value).

To identify differentially expressed genes (DEGs) between *cdh23^−/−^* and the control, we compared the transcripts per fragment kilobase million (FPKM) values of each transcript. Differential expression analysis was performed using the DESeq2 package (v1.38.3) in R. Genes with an adjusted *p* value of <0.05 and a |log_2_ (foldchange)| of ≥1 were considered DEGs. The results of DEGs were visualized with a heatmap using R package (pheatmap) (Figure 2A), where samples and related genes were clustered separately. The pink group represents the *cdh23^−/−^* group, while the blue group corresponds to the control. The results exhibited that some areas of the pink samples appear bluer, compared to the blue samples, suggesting a decrease in gene expression in *cdh23^−/−^* compared to the control.

The further visualization of the differential expression results was performed using a volcano plot generated by the ggplot2 R package (Figure 2B). A total of 1240 genes were identified as DEGs between the control and *cdh23^−/−^*, with 324 genes up-regulated and 916 genes down-regulated [27]. The volcano plot result displays the changes in differential genes, where red represents up-regulated genes, blue represents down-regulated genes, and gray dashed lines represent the threshold for selecting differential genes. The selection criteria include a significance level line at the *p* value of 0.05 and a significant vertical line at the |log_2_ (foldchange)| of 1, clearly showing the distribution of significantly differentially expressed genes.

**Figure 3:**
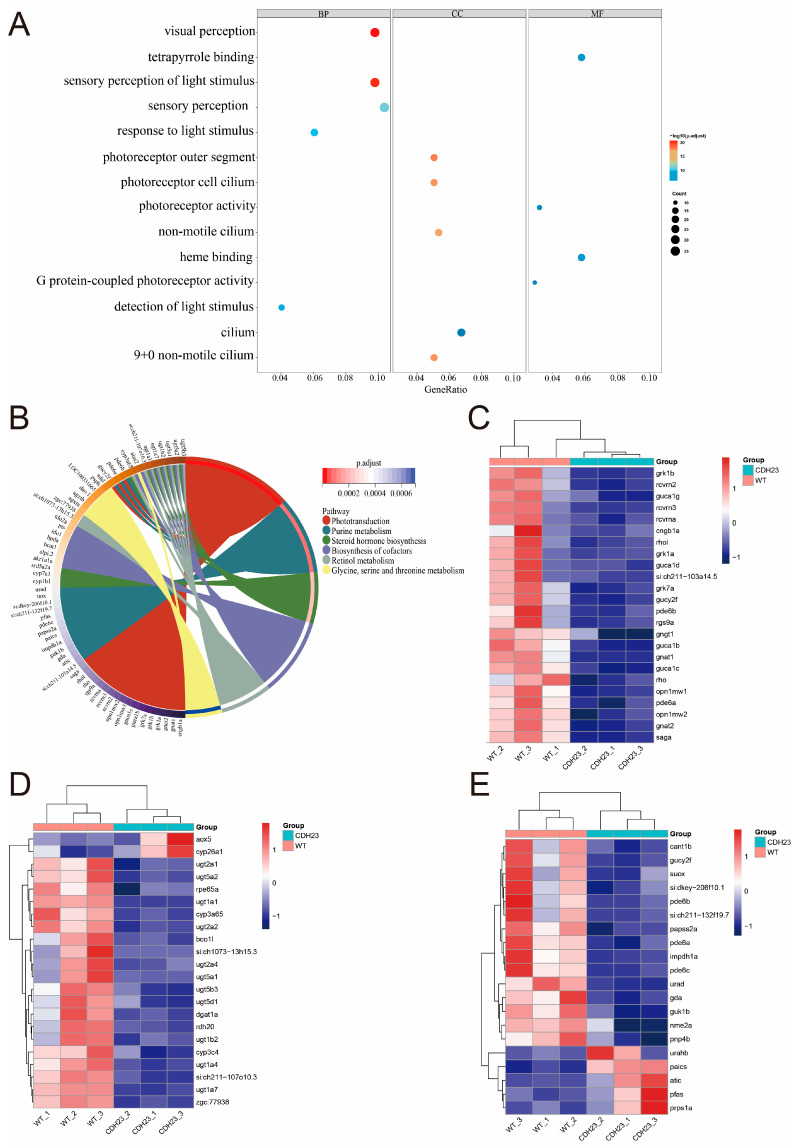
Pathways and differential gene expression associated with *cdh23* knockout in relation to photoreceptor degeneration. (**A**) Gene Ontology (GO) enrichment analysis of differentially expressed genes was conducted across three categories: biological process (BP), cellular component (CC), and molecular function (MF). The *y*-axis represents enrichment of each GO term, with redder color indicating higher significance, and size of circle representing number of genes enriched in pathway, with larger circles indicating higher number of enriched genes. (**B**) KEGG pathway enrichment analysis of differentially expressed genes was also performed, with *p*-adjust value used to assess the statistical significance of each pathway. Smaller *p*-adjust values indicate greater significance of enrichment. (**C**–**E**) Heatmap illustrating differential expression of genes involved in phototransduction, retinol metabolism, and purine metabolism pathways is presented. Vertical axis represents genes, and horizontal axis represents sample groups and control groups, with red indicating up-regulated genes and blue indicating down-regulated genes.

The Gene Ontology (GO) database is a comprehensive biological resource that links gene lists to defined terms, thereby facilitating the functional enrichment analysis of genes in the contexts of biological processes (BPs), cellular components (CCs), and molecular functions (MFs) [48]. Subsequently, the GO functional annotation was performed on differentially expressed genes, resulting in a total of 251 ontology terms, including 152 biological processes (BPs), 13 cellular components (CCs), and 87 molecular functions (MFs). In this study, we investigated whether there were abnormalities in *cdh23*-knockout embryos; thus, we focused on terms related to the visual function. Through the DEG-enriched GO analysis, we identified a total of fourteen biological processes (BPs), molecular functions (MFs), and cellular components (CCs) significantly associated with zebrafish vision and plotted the corresponding GO enrichment plots. The analysis results (Figure 3A) revealed the significant enrichment of BP terms for visual perception, sensory perception, the detection and reception of light stimulus, and the sensory perception of light stimulus. In the CC category, the DEGs were mainly associated with parts such as “photoreceptor outer segment”, “photoreceptor cell cilium”, “cilium”, “non-motile cilium”, and “9 + 0 non-motile cilium”. Meanwhile, in the MF category, the DEGs were enriched in terms such as “tetrapyrrole binding”, “photoreceptor activity”, and “G protein-coupled photoreceptor activity”. These results underscore the close association between these terms and normal zebrafish vision, highlighting the crucial role of the photoreceptor outer segment and photoreceptor cell cilium.

To further elucidate the gene pathway that affect vision, we conducted the Kyoto Encyclopedia of Genes and Genomes (KEGG) analysis. The KEGG database provides valuable information on various biological pathways, including metabolic pathways, signal transduction, and drug-related pathways [49]. DEGs were annotated to the KEGG database, and the statistical analysis of enriched pathways was performed. Combining the results from both GO and KEGG analyses, we uncovered the mechanism through which *cdh23* mutation led to light-induced sensory nerve damage. The KEGG analysis (Figure 3B) revealed the enrichment of genes such as *rcvrn*, *pde*, *cyp26a1*, *guca1b*, and *gucy2f*, which were also identified in the GO analysis. A significant proportion of DEGs were enriched in metabolic pathways, particularly those related to vision, including “phototransduction”, “retinol metabolism”, and “purine metabolism”. These findings indicate that the downstream effects following from the loss of *cdh23* disrupted the normal vision of zebrafish, which is closely related to the expression of photoreceptor-related genes. The further evaluation of the expression of genes involved in the three aforementioned pathways (Figure 3C–E) revealed that genes associated with “phototransduction” were significantly down-regulated or not expressed. In addition, genes related to “retinol metabolism” and “purine metabolism” also exhibited a decreased or absent expression.

### 2.3. Role of Cilia in Photoreceptor Function

In photoreceptor cells, the primary cilia play a key role in transporting light-conducting molecules and membrane components, supporting the process of phototransduction. Additionally, in the retina, photoreceptor cells possess highly specialized primary cilia that are essential for light detection [50].

**Figure 4:**
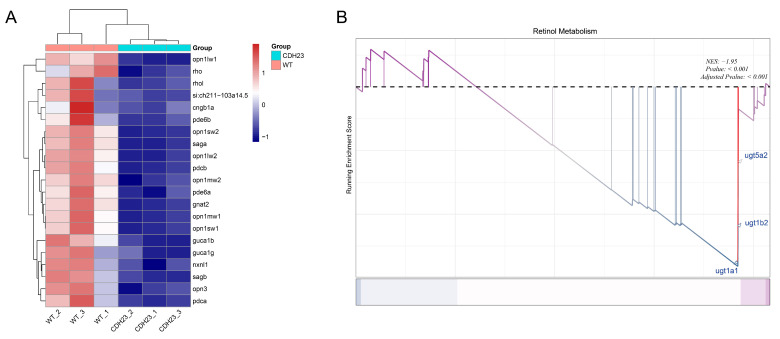
GO and GSEA. (**A**) Heatmap of differentially expressed genes in photoreceptor cilium GO enrichment pathway. (**B**) GSEA results of retinol metabolism pathway. Abnormal cGMP metabolism in *cdh23^−/−^* zebrafish retinas generates action potential in ganglion cells.

The transport of phototransduction molecules (such as rhodopsin and opsin) and membrane components between the inner segment (IS) and the outer segment (OS) is facilitated by connecting cilia, which are specialized primary cilia [51,52]. These cilia are highly specialized sensory organelles of rod photoreceptor cells, playing a crucial role in both photoreception and phototransduction processes [53,54]. In this context, we evaluated the expression of genes associated with the photoreceptor cilium pathway in photoreceptor cells. The results (Figure 4A) indicated that genes related to this pathway, such as *rho*, *pde6b*, *pde6a*, *cngb1a*, *saga*, and *sagb*, were significantly down-regulated in the *cdh23^−/−^* zebrafish model (*p* < 0.05). These findings suggest that the transport of phototransduction proteins within the photoreceptor cilium is impaired, thereby disrupting subsequent phototransduction.

### 2.4. The Precursor Substance of Rhodopsin, Retinol, Plays an Important Role in Light Conduction

Next, we conducted GSEA on all genes in the zebrafish samples from the control and *cdh23^−/−^*. The results showed that the enrichment score (ES) for the retinol metabolism pathway peaked at the tail end and had an ES value greater than 0, suggesting that the gene expression associated with this pathway was mainly present in the control. Additionally, the overall gene expression pattern for this pathway was down-regulated in *cdh23^−/−^* (Figure 4B), such as for *ugt5a2*, *ugt1b2*, and *ugt1a1*. This result is consistent with the KEGG pathway enrichment analysis (Figure 3B) and gene expression evaluation analysis (Figure 3D), indicating that *cdh23* interacts with the above genes, regulating the normal levels of retinylidene chromophore metabolism in zebrafish’s retina. The metabolite of retinol (vitamin A), 11-cis retinal, is the first relevant point of contact between the photon stimuli and the visual system [55,56,57]. It serves as the chromophore for rod opsins in the photoreceptor outer segments [58]. In rod photoreceptors, rhodopsin serves as the primary visual pigment, formed by the conjugation of 11-cis-retinal and opsin proteins [59]. This complex is essential for initiating the phototransduction cascade. The capture of a photon by the chromophore in rhodopsin induces the isomerization of 11-cis-retinal to all-trans-retinal, accompanied by a conformational change in the protein. This process ultimately leads to the activation of the downstream phototransduction cascade [60]. Rhodopsin is essential for the visual process. Even minor errors in the processes of gene transcription, translation, folding, or delivery to the designated location can lead to vision impairment [61,62,63]. The regeneration of retinol is a crucial step in visual adaptation, as it directly affects the regeneration of rhodopsin.

In the absence of *cdh23*, gene expression related to retinol metabolism appears to be down-regulated, ultimately impairing this pathway.

### 2.5. Abnormal cGMP Metabolism in cdh23^−/−^ Zebrafish Retinas Generates Action Potentials in Ganglion Cells

**Figure 5:**
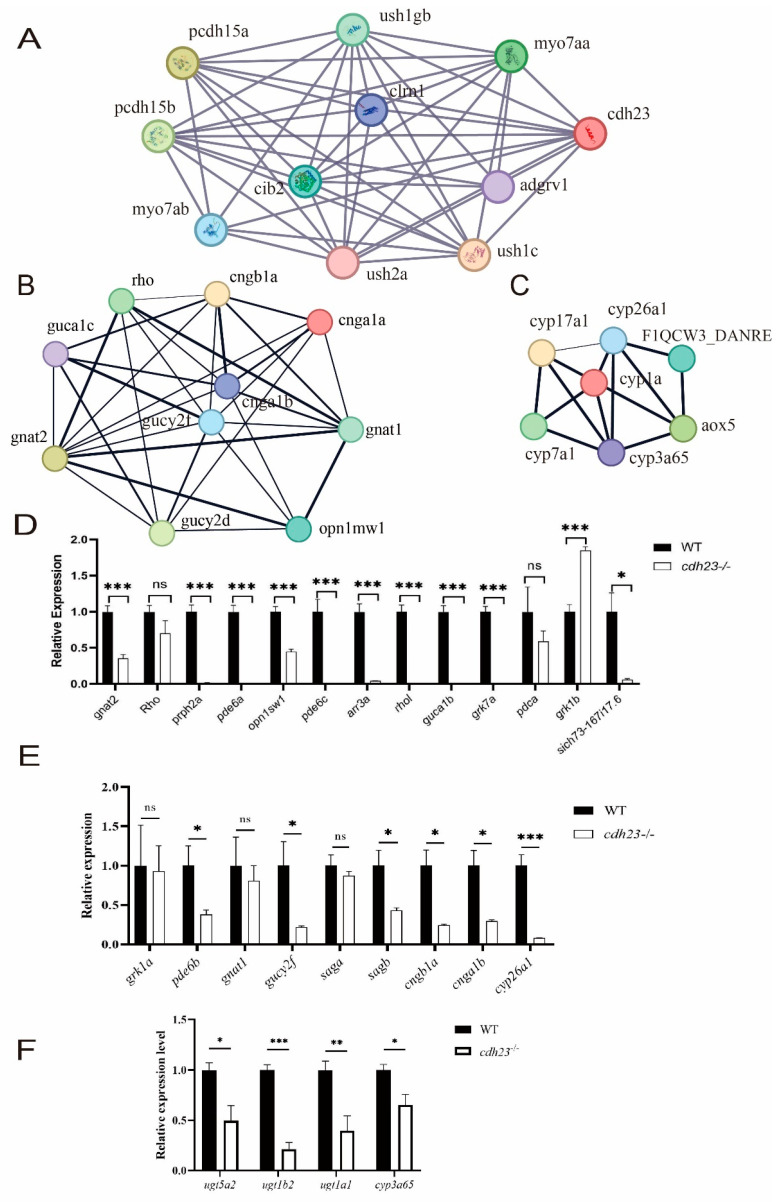
Visualization of network construction analysis of specific pathways. (**A**) PPI network representing *cdh23* gene shows proteins as nodes in graph, with their interactions depicted by lines connecting them. Various colored lines indicate different types of interactions, with thicker lines representing stronger interactions. (**B**,**C**) Key genes involved in optical conduction and retinol metabolism, as well as their interactions. (**D**,**E**) Gene expression analysis of light sensor degeneration-related genes using RT-qPCR. Additionally, statistical significance was determined with * representing a *p* value of <0.05, ** representing a *p* value of <0.01, and *** representing a *p* value of <0.001. (**F**) RT-qPCR expression analysis of genes related to retinol metabolism pathway was conducted. The results showed significant differences in gene expression levels (*: *p* value < 0.05; **: *p* value < 0.01; ***: *p* value < 0.001), indicating potential regulatory roles of these pathways in studied biological processes. ns: not significant.

To investigate the molecular mechanism of photoreceptor damage in *cdh23^−/−^* zebrafish retinas, we created a sub-regulatory network centered on *cdh23* using the STRING online platform (https://cn.string-db.org/) (accessed on 11 March 2024) (Figure 5A). Differentially expressed genes (DEGs) were utilized for constructing and visualizing the network. Key functional modules were further analyzed with the Cytoscape plugin MCODE (version = 3.9.0) (Figure 5B,C). The analysis results revealed that, in the absence of *cdh23*, the co-expressed genes *rho*, *gnat1*, *gucy2f*, *cngb1a*, and *cnga1b* play crucial roles in the phototransduction pathway of photoreceptor cells. Notably, *gnat1* encodes the α subunit of the G protein transducin, which interacts with rhodopsin in rod cells and participates in the G protein-coupled receptor pathway activated following rhodopsin stimulation [64]. The genes *cngb1a* and *cnga1b* encode the α and β subunits of the CNG channel in rod cells [65], while *cyp3a65* is involved in retinol metabolism. The loss of the *cdh23* gene leads to a cascade of peripheral reactions that perturb cGMP metabolism. These genes form a complex regulatory network that governs the binding of cGMP to the CNG channel, thereby influencing intracellular Ca^2+^ concentrations. Ultimately, these alterations prevent the generation of action potentials in ganglion cells, impairing neurotransmitter release and disrupting the phototransduction process.

We performed the enrichment analysis of genes that were significantly associated with metabolic pathways using Cytoscape. Specifically, we utilized CytoHubba to identify hub nodes. To validate these findings, we conducted RT-qPCR on the genes most closely associated with phototransduction. We collected wild-type and *cdh23*-mutant embryos on the third day and used the RT-qPCR technology with ef1a as a reference gene to detect the gene expression levels (Figure 5D–F).

Rhodopsin, a visual pigment, plays a crucial role in initiating light transduction. The *Rho* and *Rhol* genes, similar to human rhodopsin, were down-regulated, as confirmed using RT-qPCR. Additionally, several key genes in the retinol metabolism pathway, such as *Ugt5a2*, *Ugt1b2*, *Ugt1a1*, and *Cyp3a65*, have been validated to be down-regulated using RT-qPCR. *Grk1a* and *Grk7a* enable the photoreceptor activity and rhodopsin kinase activity [66]. Upon light exposure, rhodopsin binds to a downstream G protein-coupled receptor (GPCR), activating the G protein and initiating the intracellular signaling pathway [64]. The down-regulated expression of *Grk1a* and *Grk7a* was also confirmed using RT-qPCR. *Guca1b* has a calcium-binding activity and a calcium-sensitive guanylate cyclase activator activity, thereby stabilizing cytoplasmic cGMP concentrations and maintaining the opening of cGMP-gated channels [67]. The down-regulation of *guca1b* was confirmed using RT-qPCR. In the rod cells of the retina, visual signal transduction involves the activation of photosensitive pigments and the regulation of cGMP levels. *Pde6a* and *pde6b* encode the α and β subunits of a specific phosphodiesterase in rod photoreceptors, which play a crucial role in catalyzing cGMP hydrolysis [68,69]. The down-regulation of *pde6a* and *pde6b* was confirmed by RT-qPCR. The knockout of the *cdh23* gene resulted in the down-regulation of *Rho*, *Rhol*, *Ugt5a2*, *Ugt1b2*, *Ugt1a1*, *Cyp3a65, Grk1a*, *Grk7a*, *Gnat1*, *Guca1b*, *Pde6a*, and *Pde6b* genes, which is consistent with the transcriptome analysis of DEGs. The decreased expression of these genes causes elevated cGMP levels, a prolonged CNG channel opening, and an increased influx of Ca^2+^, resulting in abnormal phototransduction in photoreceptor cells and impaired vision in *cdh23^−/−^* zebrafish. Additionally, the significant down-regulation of *pde6c*, which encodes the αsubunit of cone photoreceptor phosphodiesterase [70], suggests that cone photoreceptor function is also impaired in the absence of *cdh23*. Knocking out the *cdh23* gene disrupts various components of this network, leading to complex interactions and regulations.

### 2.6. Expression of Ca^2+^ Pathway Plays Crucial Role in Photoreceptor Degeneration and Apoptosis Pathway Activation

**Figure 6:**
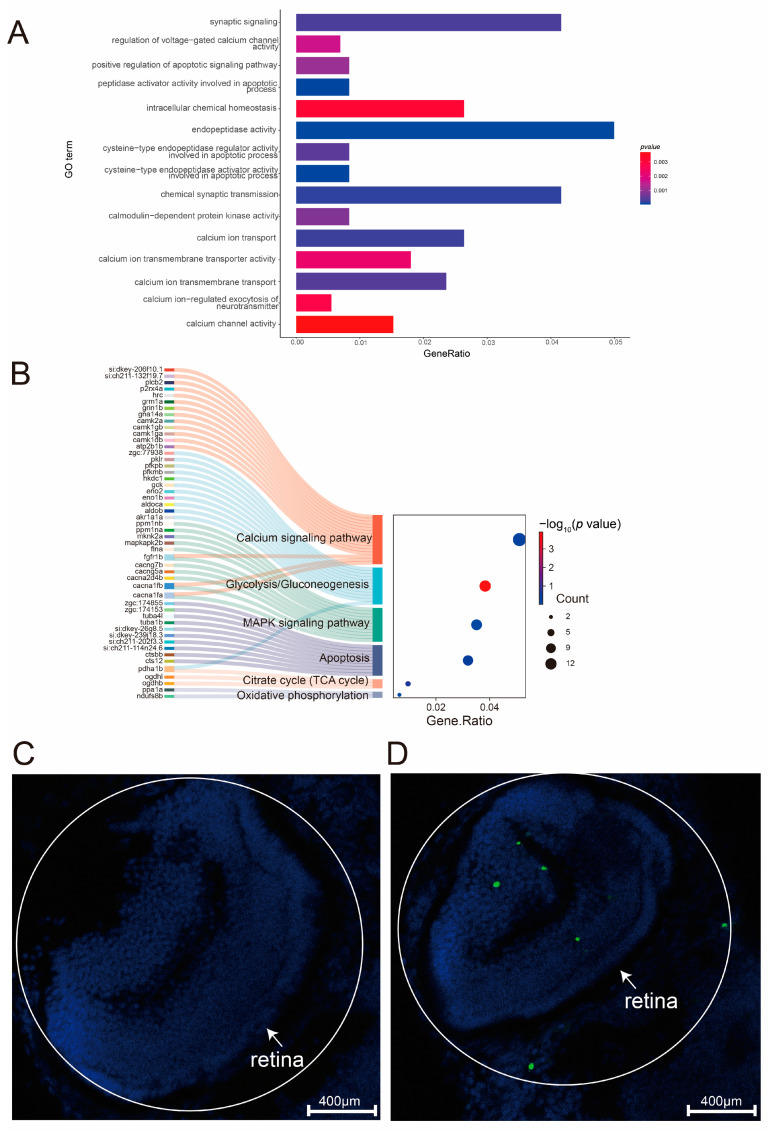
Enrichment analysis of differentially expressed genes in *cdh23^−/−^* reveals expression of Ca^2+^ pathway and cell apoptosis-related pathways. (**A**) GO enrichment analysis reveals differential expressions. Abscissa represents Gene Ratio, which is ratio of actual enriched genes in gene set for each GO term to total number of genes. Ordinate represents various enriched GO terms. *p* value measures significance of enrichment, with smaller values indicating greater statistical significance. (**B**) Differential expression genes with KEGG enrichment. Volcano plot shows enrichment of various KEGG terms and genes enriched in each pathway. Count value represents number of genes with different enrichment results. -log10 (*p* value) value measures the significance of enrichment, with a higher value indicating greater statistical significance. (**C**,**D**) Increased TUNEL staining in *cdh23^−/−^* retinas. TUNEL staining (green) on cryosections of 3 dpf zebrafish retinas revealed apoptotic cells. Zebrafish larvae were embedded for transverse sectioning. Scale bar = 400 μm.

Previous studies have demonstrated that the accumulation of cGMP in photoreceptors during phototransduction leads to the sustained activation of CNG channels, resulting in the continuous influx of Ga^2+^ [71]. Given that intracellular Ca^2+^ levels are critical for neuronal survival, we performed Gene Ontology (GO) enrichment analysis to explore the role of elevated Ca^2+^ concentrations in photoreceptor degeneration following the knockout of the *cdh23* gene in zebrafish (Figure 6A). The results revealed significant enrichment in pathways related to “calcium transport” and “synaptic function”, in addition to those associated with phototransduction. For example, in biological processes (BPs), pathways related to calcium include “calcium ion transport”, “calcium ion transmembrane transport”, the “regulation of voltage-gated calcium channel activity”, and the “calcium ion-regulated exocytosis of neurotransmitter”. However, pathways related to synaptic function are significantly enriched, such as “chemical synaptic transmission”, “synaptic signaling”, and “intracellular chemical homeostasis”. In terms of molecular function (MF), DEGs are enriched in pathways such as “calcium ion transmembrane transporter activity” and “calcium channel activity”.

In addition, apoptotic signaling pathways in zebrafish *cdh23^−/−^* rod photoreceptors were significantly regulated. Notable biological processes included the “positive regulation of apoptotic signaling pathway”, while in MF, DEGs are mainly enriched in pathways such as “endopeptidase activity”, “cysteine-type endopeptidase activator activity involved in apoptotic process”, and “cysteine-type endopeptidase regulator activity involved in apoptotic process”. Overall, most enriched terms are related to “calcium channels”, “synapses”, and “apoptosis”.

**Figure 7:**
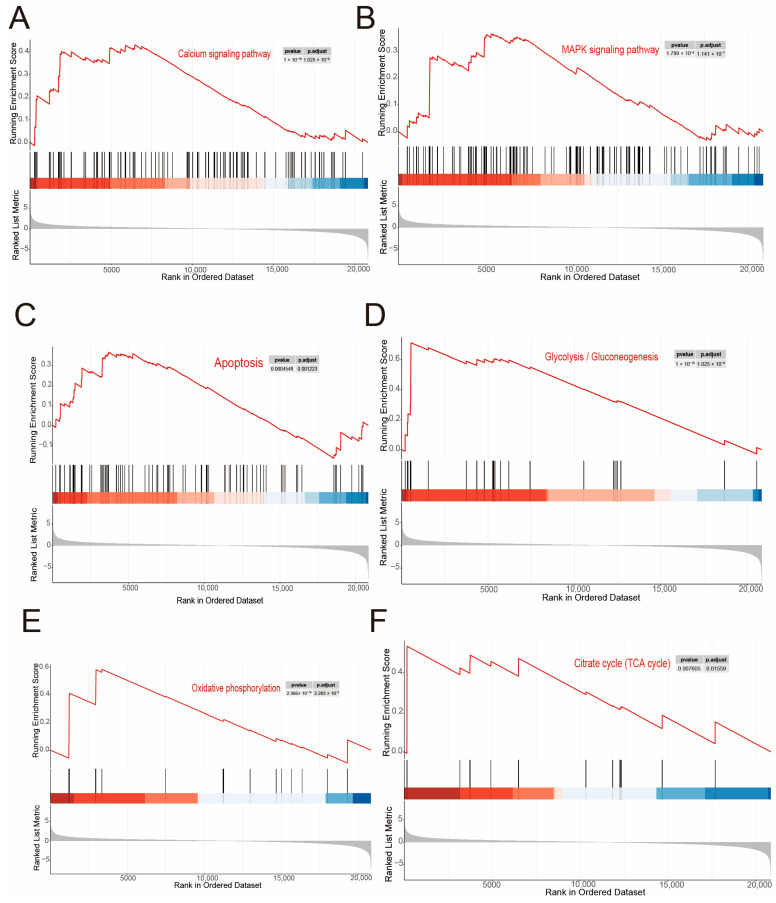
Identification of differentially expressed genes. (**A**–**F**): GSEA for calcium signaling pathway, MAPK signaling pathway, apoptosis, glycolysis/gluconeogenesis, oxidative phosphorylation, and TCA cycle.

The KEGG enrichment analysis [72] (Figure 6B) revealed that differentially expressed genes (DEGs) such as *atp2b1b*, *cacnan1fa*, *cacna1fb*, and *cts12* were significantly enriched in pathways including “calcium signaling pathway”, “MAPK signaling pathway”, and “apoptosis”. Furthermore, the GSEA revealed the expression profiles of these three pathways (Figure 7A–C), indicating an overall up-regulation of genes in the pathways of “calcium signaling pathway”, “MAPK signaling pathway”, and “apoptosis”. These findings indicate that the loss of *cdh23* gene may lead to the activation of calcium channels due to the accumulation of cGMP. Additionally, disruptions in Ca^2+^ signaling could play a role in contributing to the degeneration of rod cells.

To further evaluate if the cell death observed in the *cdh23^−/−^* retina is due to apoptosis, we performed terminal deoxynucleotidyl transferase dUTP nick end labeling (TUNEL) analysis at 3 dpf. Compared to the control, we observed an increase in cell death in the *cdh23^−/−^* retina (Figure 6C,D).

It is known that the retina has one of the highest metabolic rates in the body, which require substantial energy to maintain [73]. However, recent studies have shown that the retina predominantly relies on relatively inefficient aerobic glycolysis for energy production. Our findings indicate that energy metabolism pathways in photoreceptors, including “glycolysis/gluconeogenesis”, “oxidative phosphorylation”, and the “citrate cycle (TCA cycle)”, are significantly up regulated. Additionally, GSEA (Figure 7D–F) also revealed an overall up-regulation in these energy metabolism pathways. These results suggest that the increased influx of Ca^2+^ may indicate a higher energy demand to maintain Ca^2+^ homeostasis and the stability of the intracellular environment.

### 2.7. Ca^2+^-Dependent MAPK Pathway Regulates Energy Metabolism and Cell Death

**Figure 8:**
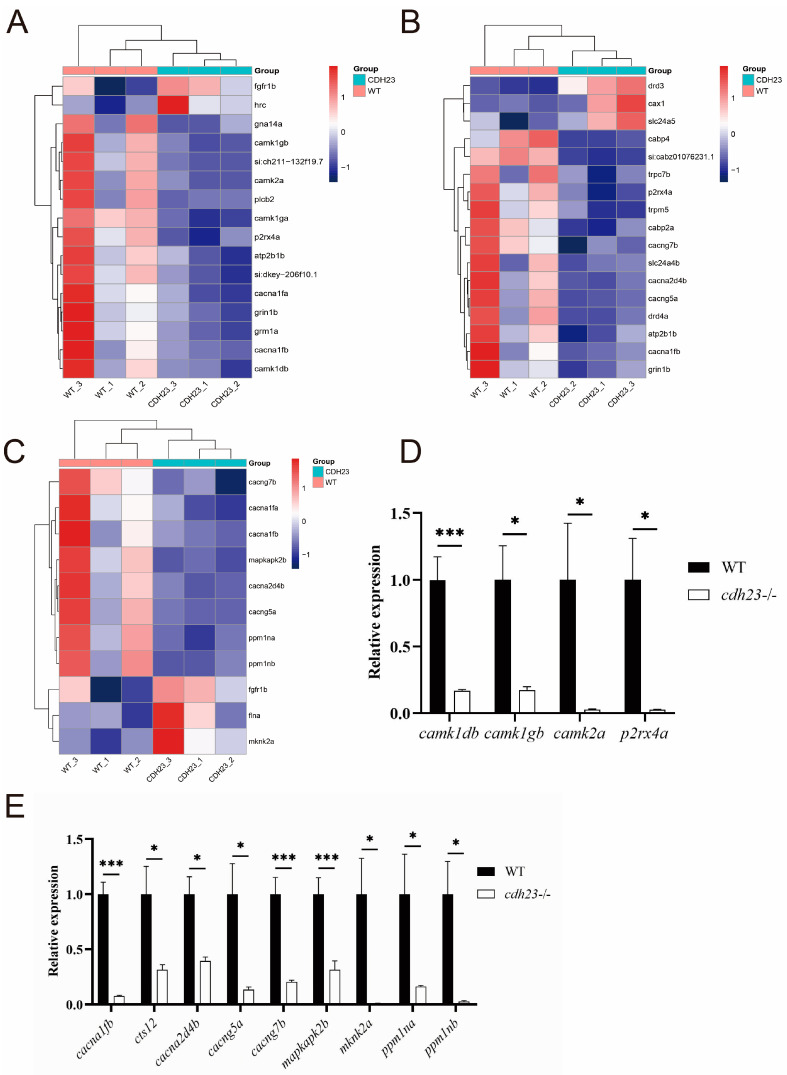
Effect of *cdh23* on calcium signaling pathway and MAPK signaling pathway. (**A**) Cluster heatmap of differentially expressed genes in calcium signaling pathway. (**B**) Cluster heatmap of differentially expressed genes in calcium ion transmembrane transport. (**C**) Cluster heatmap of differentially expressed genes related to MAPK signaling pathway. (**D**,**E**) RT-qPCR expression analysis of genes related to calcium signaling pathway and MAPK signaling pathway was conducted. Results showed significant differences in gene expression levels (*: *p* value < 0.05; ***: *p* value < 0.001), indicating potential regulatory roles of these pathways in studied biological processes.

The MAPK signaling pathway is a Ca^2+^-dependent pathway that plays a critical role in regulating cell growth, differentiation, and metabolism. The activation of calcium signaling is often associated with cell apoptosis [74]. In this study, we focused on evaluating the transcriptional changes in genes associated with the calcium signaling pathway, calcium ion transmembrane transport, and the MAPK signaling pathway in the absence of the *cdh23* gene (Figure 8A–C). Moreover, we collected wild-type and *cdh23*-mutant embryos on the third day and used the RT-qPCR technology with *ef1a* as a reference gene to detect the gene expression levels (Figure 8D,E). Our results indicated that, within the calcium signaling pathway, the expression of genes encoding the L-type voltage-gated calcium channel α1 subunit, *cacna1fb*, was down-regulated. These channels are essential for controlling intracellular Ca^2+^ influx in neural cells and require ATP for their function [75]. The qPCR results further confirmed that the expression of these genes was down regulated. Furthermore, genes encoding calcium-dependent protein kinases, such as *camk1db*, *camk1ga*, *camk1gb*, and *camk2a*, which are activated downstream in apoptosis signaling upon an increase in Ca^2+^ concentration [76], also showed decreased expression. Similarly, the *atp2b1b* gene encoding the calcium ion transport ATPase, which pumps Ca^2+^ out of the cytoplasm, also showed down-regulated expression. In addition, several genes involved in the MAPK signaling pathway, particularly those regulating voltage-gated calcium channels (VGCCs) and cyclic nucleotide-gated (CNG) channels, were significantly altered. These include *cacna1fa*, *cacna1fb*, *cacna2d4b*, *cacng5a*, and *cacng7b*, all of which showed down-regulated expression, as confirmed using qPCR. Collectively, these results indicate that, in the absence of the *cdh23* gene, both the MAPK and calcium signaling pathways play a role in regulating rod cell apoptosis.

## 3. Discussion

In this study, we used zebrafish *cdh23*-knockout lines, with the DEG transcriptome data analysis, retinal histological H&E staining, TUNEL assay, and q-PCR experiments, to explore the physiological and morphological functions of this gene. Transcriptome data comparison revealed that DEGs were significantly enriched in pathways related to the “outer segment of photoreceptor cells”, “light conduction”, “synapse”, “calcium signaling”, and “apoptosis”. Notably, genes involved in these pathways include *rho*, *gnat1*, *cngb1a*, *cngb1b*, *pde6a*, *pde6b*, *grk1a*, *guca1b,* etc. In addition, our analysis suggests that changes in Ca^2+^ signaling are closely associated with the MAPK signaling pathway.

Connecting cilium is a specialized structure located between the outer segment and the inner segment of rod cells. All the proteins located in the outer segments are synthesized in the inner segments and must traverse the connecting cilium to reach their final destination. Therefore, the connecting cilium is responsible for the intracellular transport of photoreceptor proteins, such as rhodopsin and other phototransduction proteins, in visual cells [51,52,77,78,79]. The visual signal transduction process is rapid and highly organized; a single activated rhodopsin molecule can activate approximately 800 G proteins. When the retina is exposed to illuminated light, the conformational change in rhodopsin on the membrane disks of rod cell outer segments triggers the activation of transducin, a G protein located on the membrane disks. This activation subsequently stimulates the phosphodiesterase (PDE) activity, leading to the degradation of cGMP, resulting in a decrease in its concentration [80].

The conformational changes in rhodopsin involve the decomposition and regeneration of retinol, which is the first and crucial step of photoconduction [59,60]. In this study, transcriptome data analysis indicated that normal visual signal transduction depends on photoreceptor cells. ATP, the primary energy currency in cells, provides the energy required for various biological processes within the cell. In rod cell photoreceptors, ATP is essential for the movement of cilia, which are crucial for sensing light stimuli and transmitting visual signals. Furthermore, processes such as the activation of G protein-coupled receptors and the initiation of secondary signal pathways also require ATP as the primary energy source. Previous studies have shown that purine metabolism is disrupted in *cdh23^−/−^* individuals, which leads to ATP deficiency [36].

Rhodopsin, also known as visual purple, is the most abundant protein in rod cells. Most mutations in rhodopsin are caused by defects in proper folding or transport to the outer segment, resulting in autosomal dominant retinitis pigmentosa (RP) [74]. This study confirms that the expression of the *Rho* and *Rhol* genes is decreased, indicating that the absence of the *cdh23* gene may impair the normal function of the photoreceptor cilia. This, in turn, affects the proper localization and regeneration of rhodopsin in the outer segment.

In rd1 mice, rod photoreceptor loss occurs through a non-apoptotic cell death mechanism that is driven by elevated intracellular cGMP levels [21]. Transcriptional data analysis has revealed that down-regulated genes, including *gant1*, *grk1a*, *pde6a*, and *pde6b*, which are involved in the metabolism process, likely contribute to the accumulation of cGMP in the phototransduction cascade. This accumulation activates the CNG channel, leading to prolonged channel opening, which results in an increased influx of Ca^2+^ and Na^+^ [71]. The sustained depolarization due to CNG channel activity further triggers the persistent activation of voltage-gated calcium channels (VGCCs) at the synaptic terminals of photoreceptors, which increases the influx of Ca^2+^ and accelerates photoreceptor degeneration [75]. Membrane potential changes triggered by the activity of CNGC or VGCC may affect the activity of sodium–calcium exchangers (Na^+^/Ca^2+^ exchangers, NCX), leading to the influx of extracellular Ca^2+^ through NCX [81].

The precise regulation of intracellular Ca^2+^ levels is essential for neuronal survival [82]. Correspondingly, Ca^2+^ channels have been extensively studied as potential therapeutic targets for retinal degeneration (RD) over the past two decades. The prevailing hypothesis is that the excessive activation of Ca^2+^ channels leads to intracellular Ca^2+^ overload, triggering the death of photoreceptor cells [83,84]. In this study, we observed significant changes in the regulation of key processes, including “calcium ion transport”, “calcium ion transmembrane transport”, “calcium ion-regulated exocytosis of neurotransmitter”, “calcium ion transmembrane transporter activity”, and “calcium channel activity” following the deletion of the *cdh23* gene. Notably, the expression of *cngb1a* and *cnga1b*, which encode the α and β subunits of CNG channels in rod cells, was down regulated. Similarly, the expressions of *cacna1fa* and *cacna1fb*, which encode VGCC subunits, was also reduced. We hypothesize that the down regulation of these genes represents a compensatory mechanism aimed at maintaining normal visual function. However, the expression of the gene *slc24a5*, which encodes the NCX reverse transporter [85], was up-regulated, potentially resulting in an influx of Ca^2+^. Moreover, genes encoding downstream calcium-dependent proteases, such as *camk1db*, *camk1ga*, *camk1gb*, and *camk2a*, also showed that CAMK is a key signaling molecule that mediates the indirect regulation of the MAPK pathway by calcium signals [76].

*atp2b1b*, a gene encoding a calcium ion-transporting ATPase belonging to the *p*-type ATPase family, plays an essential role in extruding calcium ions from the cytoplasm to sustain the calcium gradient across the cell membrane. This process is essential for regulating the spatiotemporal distribution of calcium signals. In photoreceptor outer segments, ATP2B1b is crucial for maintaining Ca^2+^ homeostasis during phototransduction. The inhibition of ATP generation due to defects in purine metabolism may impair the activity of this calcium ion pump, potentially enhancing Ca^2+^-dependent MAPK signaling.

Therefore, to a certain extent, the expression of the MAPK signaling pathway was found to progressively increase after the knockout of the *cdh23* gene. Additionally, DEGs were significantly enriched in pathways associated with intracellular enzyme activity related to cell apoptosis, and the TUNEL assay results showed that there were apoptotic signals in the *cdh23^−/^^−^* retina. These findings suggest that, during the late stages of rod cell degeneration, the reduced survival of rod cells may contribute to the decline in zebrafish visual ability. Additionally, the results indicated that cone cell photoreceptor activity was also partially impaired.

**Figure 9:**
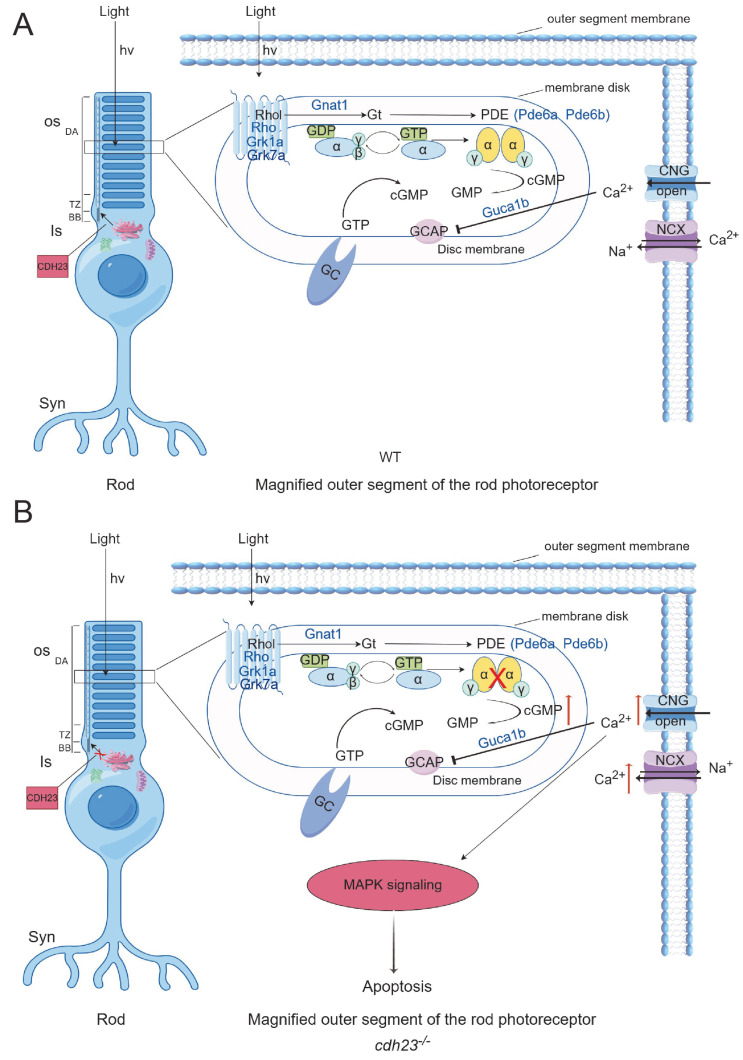
Schematic diagram of *cdh23* regulating rod cell calcium ion transport (image created by Figdraw). (**A**) In WT rod photoreceptor cells, genes such as *Rho*, *Rhol, Grk1*, Grk7a, *Gnat1*, *Pde6a*, *Pde6b*, and *Guca1b* play essential roles in phototransduction. Additionally, Atp2b1b is responsible for transport of calcium ions (Ca^2+^). Rhodopsin is synthesized in endoplasmic reticulum of inner segment (IS) and transported to outer segment (OS) through vesicular transport from Golgi apparatus, with IS and OS being connected by connecting cilium. Purine metabolism generates sufficient ATP to properly position rhodopsin in outer segment. Upon light stimulation, rhodopsin undergoes conformational change, activating Gt transducin protein, which subsequently activates phosphodiesterase (PDE). PDE hydrolyzes cGMP, leading to decrease in its concentration, which causes closure of CNG channels. This reduces inward flow of Ca^2+^ and release of neurotransmitter glutamate, completing phototransduction process. (**B**) In *cdh23^−/−^* embryos, disrupted purine metabolism leads to insufficient ATP production, impairing vesicle transport from Golgi apparatus and movement of cilium. As a result, rhodopsin fails to localize properly to outer segment, preventing cGMP hydrolysis by PDE. Increased cGMP concentration activates CNG channels, causing continuous inward flux of Ca^2+^, which activates Ca^2+^-dependent MAPK signaling pathway and triggers apoptosis in rod photoreceptor cells.

Retinal energy metabolism is known to be highly energy-intensive and metabolically active, primarily due to the need to maintain ion homeostasis within retinal cells. In rd mice, the influx of Ca^2+^ is dependent on the inefficient aerobic glycolysis in the retina, which converts glucose to pyruvate and subsequently to lactate under aerobic conditions, rather than undergoing more efficient mitochondrial oxidation. Otto Warburg first discovered this phenomenon in the early 1920s, naming it the “Warburg effect” [79]. However, the specific reasons for this mechanism are still under investigation. In this study, an apparent enhancement in the activity of the “oxidative phosphorylation” pathway was observed, which we speculate may be related to rod cell apoptosis (Figure 9).

## 4. Materials and Methods

### 4.1. Data Collection

Six datasets were obtained from the Gene Expression Omnibus (GEO) database [36] (accession number: PRJNA904934), including differentially expressed genes (DEGs) in *cdh23^−/−^* and the control (72 h embryo tissue), with each group replicated three times. Detailed information about these datasets is provided in the Supplementary Materials.

### 4.2. RNA-Seq Data Analysis

The quality of raw reads was evaluated by the software FastQC (v 0.11.5). The software Trimmomatic (v 0.36) software used to remove adapters and poor-quality reads. After this step, the clean reads were aligned to the GRCz11 reference genome using the software Hisat2. The alignment results were subsequently quantified for gene expression using HTSeq-count (v 0.9.1). Principal component analysis (PCA) was performed and visualized using R package to show the clustering of the two datasets. Differentially expression analysis was performed in R software using Deseq2 (v 1.38.3) package, with genes exhibiting an adjusted *p* value of <0.05 and a |log_2_ (fold change)| of ≥1 considered as differentially expressed genes (DEGs). The results of differential expression were visualized using heatmaps generated with the R package pheatmap, and a volcano plot was used to depict the DEGs.

### 4.3. Functional Enrichment Analysis

DEGs identified between the control and the *cdh23*-knockout group were subjected to Gene Ontology (GO) and Kyoto Encyclopedia of Genes and Genomes (KEGG) enrichment analyses using the Cluster Profiler (v 4.7.1.003) package. An adjusted *p* value of <0.05 was considered as statistically significant.

### 4.4. PPI Network Construction and Analysis

To investigate the molecular mechanism of photoreceptor damage in *cdh23*^−/−^ zebrafish retinas, we created a sub-regulatory network centered on *cdh23* using the STRING online platform (https://cn.string-db.org/) (accessed on 11 March 2024). Differentially expressed genes (DEGs) were utilized for constructing and visualizing the network. Key functional modules were further analyzed with the Cytoscape plugin MCODE (version = 3.9.0), applying the following selection criteria: K-core = 2, degree cutoff = 2, max depth = 100, and node score cutoff = 0.2. This approach enabled the identification of key subnetworks and genes based on the relationships between edges and nodes, which were then selected for the subsequent KEGG enrichment analysis. Subsequently, we performed the enrichment analysis of genes significantly associated with metabolic pathways using Cytoscape. Specifically, we utilized CytoHubba, a network topology analysis plugin, which applies various algorithms to identify hub nodes, such as Degree, BottleNeck, and Closeness, etc. In this study, we utilized the Degree algorithm, which evaluates nodes based on their degree value, specifically the number of connections each node possesses within the network.

### 4.5. Cryosection

The control and *cdh23*^−/−^ zebrafish embryos at 3 dpf were collected separately. The embryos were fixed overnight at 4 °C in freshly prepared 4% paraformaldehyde (PFA). Subsequently, the fixed embryos were embedded in the OTC compound (Tissue-Tek) and rapidly frozen in liquid nitrogen. Cryosections with a thickness of 10 μm were then prepared using a cryostat (Leica, Wetzlar, Germany). From the obtained serial sections, samples were randomly selected for the TUNEL assay.

### 4.6. Paraffin Section

Wild-type embryos and *cdh23*^−/−^ embryos of 3 dpf zebrafish were collected and fixed overnight with 4% paraformaldehyde prepared fresh at 4 °C. Then, they were washed with distilled water to remove the fixative residue. The material was then dehydrated stepwise in an ethanol solution. After the dehydration was complete, the tissue was removed for embedding. The wax block was frozen on a frozen table for several minutes, and the frozen wax block was put into the paraffin slicer. The angle and thickness of the slicer were adjusted, the slice size was 3–5 microns, and the cut paraffin sheet was rolled with a brush to flatten the sheet and then scoop the sheet out. Then, samples were randomly selected for hematoxylin and eosin staining.

### 4.7. Hematoxylin and Eosin Staining

First, the paraffin sections were dewaxed with water, and the paraffin sections were soaked in xylene I for 20 min, xylene II for 20 min in turn, and then in anhydrous ethanol I, anhydrous ethanol II, and alcohol III for 5 min each, and finally, they were rinsed with tap water. The hematoxylin staining of the paraffin sections was performed. The paraffin sections were placed in the hematoxylin dye solution for 3–5 min, and the excess dye solution was washed with tap water, differentiated with hematoxylin differentiation solution for a few seconds, rinsed with tap water, stained with hematoxylin blue solution for a few seconds, and finally rinsed with running water for a few seconds. Then, the eosin staining of the paraffin sections was performed; the slices were dehydrated in 85% ethanol and 95% ethanol successively, and then stained with eosin staining solution for 5 min. Finally, the dewatering of the slices was carried out.

### 4.8. TUNEL Assay

The control and *cdh23*^−/−^ embryos were collected at 3 days post-fertilization (3 dpf). The embryos were fixed in 4% paraformaldehyde (PFA) at 4 °C overnight. Following this fixation, the embryos were washed with PBST; then, the tissues was dehydrated in 30% sucrose at 4 °C for cryoprotection. Then, the cryosections of these tissues were made. For the detection of apoptosis in retinal cells, the cells were analyzed using the In Situ Cell Death Detection Kit, Fluorescein, according to the manufacturer’s instructions.

### 4.9. Quantitative Real-Time Reverse Transcription Polymerase Chain Reaction (RT-qPCR)

PCR was performed using the StepOne Plus Real-Time PCR System (Thermo Fisher Scientific, Waltham, MA, USA), and its mix contained the cDNA template, primers, and TB Green™ Fast qPCR Mix (TaKaRa, Kusatsu, Japan). The relative expression levels of target genes were determined by comparing with the standard curve.

### 4.10. Zebrafish Maintenance

This study used zebrafish (TU) from the Laboratory of Animal Nutrition and Human Health, School of Life Sciences, Hunan Normal University. Zebrafish embryos were obtained through natural mating and maintained at 28.5 °C in fish facilities. To inhibit pigmentation, embryos were treated with 0.2 mM 1-phenyl-2-thiourea (PTU). When embryos reached the desired developmental stage, they were then collected and fixed overnight at 4 °C in 4% paraformaldehyde (PFA) in phosphate-buffered saline (PBS).

## Data Availability

The datasets presented in this study can be found in online repositories. The names of the repository/repositories and accession numbers are as follows: https://www.ncbi.nlm.nih.gov/ (accessed on 3 December 2023); PRJNA904934, SAMN31858593, SAMN31858594, SAMN31858595, and SAMN31858596.

## Supplementary Materials

The following supporting information can be downloaded at: https://www.mdpi.com/article/10.3390/ijms26104604/s1.
